# Strong and weak points in the quality of life of school-age children with newly diagnosed uncomplicated epilepsy over the first six months: golden hours for prevention

**DOI:** 10.3325/cmj.2024.65.349

**Published:** 2024-08

**Authors:** Željka Rogač, Dimitrije Nikolić, Aleksandar Dimitrijević, Ivana Andrić, Goran Milošević, Dejan Stevanović

**Affiliations:** 1 Clinical Centre of Montenegro – Institute for Children’s Diseases, Podgorica, Montenegro; 2 Faculty of Medicine, University of Montenegro, Podgorica, Montenegro; 3 Medical Faculty, University of Belgrade, Belgrade, Serbia; 4 University Children's Hospital, Belgrade, Serbia; 5 Faculty of Medical Sciences, University of Kragujevac, Kragujevac, Serbia; 6 Clinic for Neurology and Psychiatry for Children and Youth, Belgrade, Serbia

## Abstract

**Aim:**

To determine changes in the quality of life associated with epilepsy in school-age children with newly diagnosed uncomplicated epilepsy over the first six months after diagnosis to find points relevant for the early prevention of deterioration in quality of life.

**Methods:**

This prospective follow-up study, performed in University Children’s Hospital in Belgrade, enrolled 60 school-aged children with recently diagnosed epilepsy, along with their parents. The respondents completed the Children with Epilepsy Quality of Life immediately following the diagnosis of epilepsy and six months later.

**Results:**

Significant decline was observed in the domains related to intrapersonal/emotional relationships by both children (*P* < 0.001) and their parents (*P* = 0.03), and in the need to keep epilepsy a secret as observed by parents (*P* = 0.04). Significant improvement was found in the Interpersonal/Social domain as rated by parents (*P* = 0.001). Total quality-of-life scores, as assessed by children and parents, did not change significantly.

**Conclusion:**

Bearing in mind that stigma and intrapersonal struggles are the major factors affecting the quality of life in children with epilepsy, psychological and social support is highly recommended in the first six months following an epilepsy diagnosis. Since intrapersonal relationships improved over six months, compensating for other deteriorations in the quality of life, children with epilepsy should be encouraged to socialize with their peers and to join organizations and actions that encourage social contact.

The World Health Organization defines quality of life (QOL) as “an individual's perception of their own life in the context of the culture and system of values in which they live, and in relation to their own goals, expectations, standards, and interests” (1). It is a broad concept influenced by many factors, such as an individual's health, mental state, degree of independence, social relationships, and, most importantly, environmental factors, changes, and occurrences (2). The diagnosis of epilepsy, especially in economically underdeveloped areas, is shrouded in a veil of stigma that significantly affects all the mentioned domains (3). Therefore, monitoring the QOL of a child with epilepsy is demanding, and it must also be viewed in a broader context of stigma and the family's daily functioning (4,5).

Usually, poor QOL is observed in chronic patients who are treated with polytherapy and have the pharmacoresistant type of epilepsy (6). However, a one-year follow-up study of children with epilepsy showed a decline in QOL even one month after starting the treatment (7). Furthermore, difficulties in psychosocial functioning in children with newly diagnosed epilepsy appeared as early as three months after the disease onset and persisted for the next two years (8). On the other hand, a two-year follow-up study found that children with epilepsy generally had a good QOL, although there was a significant temporary decline during the first six months (9).

The present study is a continuation of two previous studies on cognitive status, behavior, anxiety and depression, and general QOL, in school-age children with recently diagnosed epilepsy in the first six months after diagnosis (10,11). We showed the occurrence of significant internalizing and externalizing symptoms after six months and a mild decline in cognitive status (10). Also, general QOL significantly deteriorated even in the first six months after the diagnosis. The main predictor of this deterioration was a significant occurrence of internalizing symptoms and a low general QOL at the beginning of the disease (10).

The general QOL is a good indicator of the psychosocial aspects of all children (9). However, since epilepsy is a specific disease associated with greater stigma than other chronic diseases, the QOL of the patients with epilepsy must be seen through a unique prism of epilepsy-related QOL, which simply considers the evaluations of the impacts of epileptic seizures and treatment regimens on various aspects of everyday functioning and well-being of the person with epilepsy (12). Previous studies emphasized the weak points in the quality of life of children with epilepsy (7-9). So, we wanted to identify the strong ones, which may be the focus of prevention activities. This is precisely the purpose of this research, carried out on the same group of children as in the previous two studies, who have newly diagnosed epilepsy and suffer from no other diseases. The study aimed to determine changes within particular domains of psychosocial functioning over the first six months after diagnosis.

## Respondents and methods

The study was designed as a prospective follow-up study of school-aged children with newly diagnosed epilepsy. It was conducted in the University Children's Hospital in Belgrade, Serbia, in 2021. Ethical approval was granted by the Ethics Committee of the University Children's Hospital in Belgrade. Written consent from parents/guardians was obtained for all participating children.

The first visit took place immediately after the introduction of anti-seizure medication (ASM) for the first time in children’s lives (average time of two days, range 0-4 days), and the second visit took place six months after therapy initiation. Patients were successively included in the research. The inclusion criteria were school-age, normal neurological and physical status, IQ>80, absence of comorbidities, idiopathic causes of epilepsy – ie, absence of structural lesions on neuroimaging of the endocranium, and genetic and metabolic causes of epilepsy. The exclusion criteria were poor compliance and the desire for alternative medications.

After providing informed consent to participate, children and parents independently and separately filled out the Health-Related Quality of Life Measure for Children with Epilepsy (CHEQOL-25). A separate form was used to gather demographic data (sex and age) and clinical data (seizure control, type of seizure, type of ASM). Seizure control was categorized as complete (no seizures during the six months of follow-up), partial (seizures in the first three months, followed by no seizures), and poor (seizures throughout all six months).

The CHEQOL-25 is specifically designed to assess the QOL in pediatric epilepsy. The CHEQOL-25 questionnaire has a self-report and parent/guardian-report version. The self-report version consists of five subscales/domains: Interpersonal/Social, Current Worries, Intrapersonal/Emotional, Epilepsy as a Secret (in the sense of stigma), and Need for Normality. Each domain contains five questions. The parent version also has five subscales/domains: Interpersonal/Social, Current Worries, Future Concerns, Intrapersonal/Emotional, and Epilepsy as a Secret. The answers to the questions are given on a Likert scale from 1 to 4, where a higher score reflects a more positive perception. The total subscale score is the sum of all the questions answered. We calculated the CHEQOL-25 total score as a global QOL related to epilepsy. It comprises all subscale questions that exhibited adequate internal consistency of measurement, specifically 18 questions in the self-report version (items 7, 16, 18, 21, 22, 23, and 25 were omitted due to low correlation with the corrected total score) and 20 questions in the parent questionnaire version (Epilepsy as a Secret subscale was omitted). The questionnaire is a reliable and valid instrument (13-15), and the Serbian version of the questionnaire was used (16). Internal consistency reliability, measured by Cronbach's α coefficient, was low for the subscales of Undisclosed Epilepsy in both versions (0.11 and 0.59), and Wish for a Normal Lifestyle (0.46) and Current Concerns (0.57) for the self-report version. For the other scores it was ≥0.77. Total scores had an α of 0.90 for self-report and 0.94 for the parent version.

### Statistical analysis

The normality of distribution was tested with the Shapiro-Wilk test. To assess the differences in the questionnaire scores at the beginning of treatment (before) and after six months of follow-up (after), a paired *t* test was used. For statistically significant differences, the effect size of the change in scores was expressed by Cohen's d coefficient and interpreted as small (<0.5), medium (0.5-0.8), or high (>0.8) (17). *P* values <0.05 were considered statistically significant. All analyses were conducted with SPSS, version 18 (SPSS Inc., Chicago, IL, USA).

## Results

The study enrolled 69 children with newly diagnosed epilepsy and their parents. Nine children were excluded before the second visit due to poor compliance and the need for polytherapy. At the second visit, general data after six months were available for 60 children (Table 1), while data on the quality of life from both of the assessments were available for 59 respondents (Tables 2 and 3).

**Table 1:** Children’s basic demographic and clinical data

	Six months follow-up, n = 60
Age, mean (SD), range	12.45 (3.25), 7-18
Male/female sex, n (%)	34 (56.7)/26 (43.3)
Seizure control, n (%)	
complete	35 (58.3)
partial	20 (33.3)
poor	5 (8.3)
Antiepileptic, n (%)	
valproate	18 (30)
levetiracetam	15 (25)
carbamazepine	13 (21,7)
lamotrigine	7 (11.7)
etosuximide	6 (10)
topiramate	1 (1.7)
Type of seizures, n (%)	
generalized	47 (78.3)
focal	7 (11.7)
focal with secondary generalization	6 (10)

*SD – standard deviation.

**Table 2:** Children with Epilepsy Quality of Life-25 self-reported scores at baseline and six-month follow-up in children (N = 59)

Subscale	Visit, mean (standard deviation)		t (p value)
	baseline	six-months	
Interpersonal/Social	16.62 (3.65)	16.83 (3.87)	-0.38 (0.70)
Current Worries	12.11 (3.16)	11.82 (3.21)	0.61 (0.55)
Intrapersonal/Emotional	15.78 (3.15)	13.67 (3.63)	3.82 (<0.001)
Epilepsy as a Secret	13.35 (2.39)	13.67 (1.78)	-0.91 (0.37)
Need for Normality	10.02 (2.44)	9.82 (2.51)	0.57 (0.57)
Total score	54.85 (10.519)	53.25 (10.23)	1.01 (0.29)

**Table 3:** Children with Epilepsy Quality of Life-25 scores at baseline and six-month follow-up in parents (N = 59)

Subscale	Visit, mean (standard deviation)		t (p value)
	baseline	six-months	
Interpersonal/Social	15.70 (3.95)	17.33 (3.30)	-3.47 (0.001)
Current Worries	12.28 (3.52)	13.17 (3.30)	-1.92 (0.06)
Future Concerns	14.93 (3.90)	16.12 (3.49)	-2.46 (0.02)
Intrapersonal/Emotional	14.33 (4.03)	13.12 (3.69)	2.21 (0.03)
Epilepsy as a Secret	11.53 (2.92)	10.73 (2.73)	2.06 (0.04)
Total score	57.25 (13.05)	59.73 (11.16)	-1.73 (0.09)

Children reported a significant decrease on the Intrapersonal/Emotional subscale (*t* = 3.82; *P* < 0.001; d = 0.53; Table 2). Parents, on the other hand, reported small, yet significant, improvements in the domains of Interpersonal/Social (*t* = -3.47; *P* < 0.001; d = 0.41) and Future Concerns (*t* = -2.46; *P* = 0.02; d = 0.29). Parents also observed small, yet significant, worsening on the Intrapersonal/Emotional (*t* = 2.21; *P* = 0.03; d = 0.28) and Epilepsy as a Secret (*t* = 2.06; *P* = 0.04; d = 0.26; Table 3) scale. The total scores on quality of life did not change significantly for either.

## Discussion

The present study demonstrated that the mere diagnosis of epilepsy itself could have some negative effects on the everyday life and QOL of children with uncomplicated epilepsy within the first six months. During this period, children reported a significant deterioration in emotional functioning and, according to their parents, an increased sense of stigma. On the other hand, social functioning and concerns about the future improved. Nevertheless, although we previously reported a significant decline in the general QOL within the same group of patients (10), the QOL associated with epilepsy did not change significantly.

Both interpersonal and emotional functioning, as reported by children and their parents, significantly declined within the first six months following diagnosis. Previous research has shown that emotional functioning and stability in children with epilepsy are significantly impaired, particularly in patients receiving polytherapy and those having poor seizure control and chronic epilepsy (18). Therefore, the finding that interpersonal competencies, which are a significant component of the QOL of children with epilepsy, become significantly compromised within the first six months underscores the need for timely psychological support for these children (19). Recently, the International League Against Epilepsy (ILAE) has issued recommendations for the pediatric population regarding intrapersonal competence disorders, which may involve active psychological support and the involvement of a child psychiatrist (20,21).

According to the current findings, children with newly diagnosed epilepsy after six months show a desire to lead a normal life, a finding that has also been observed previously (22). A qualitative study (23) interviewing children with epilepsy found that they aspired to be like their peers without limitations in performing everyday activities. Despite the mentioned study being conducted around 20 years ago, the same issue remains relevant. Hence, pediatricians should inform children with epilepsy that they are permitted to perform most age-appropriate activities (24). For instance, according to ILAE recommendations, individuals with epilepsy may participate in most professional sports (5). Encouraging the establishment of an Epilepsy Association may help foster healthy lifestyles and self-help among patients' families, empowering them to take pride in their struggle with the disease (25,26).

However, according to parents in our study, the trend of stigma significantly increased during the follow-up period, even though the children themselves did not attach as much importance to it. Therefore, the implementation of the activities mentioned above may prove challenging. Given that in traditional societies, such as Serbian, stigma arises due to fear of the unknown, health managers should aim to educate the general population about the symptoms, origins, and consequences of epilepsy (27). Furthermore, a recent cross-sectional study demonstrated that children with higher seizure self-efficacy experienced fewer problems with stigma and enjoyed better QOL (28). Thus, educating children with epilepsy, which falls within the purview of competent epileptologists, can also reduce the stigma (29).

Encouragingly, parents were significantly more optimistic about their children's future after six months than at baseline. This result differs from previous findings and may be attributed to the favorable seizure control during the first six months, which was complete in more than half of our respondents (30). Nevertheless, the parents of children with long-standing epilepsy tend to negatively respond to similar questions, such as those regarding future career choices. These findings emphasize the need to nurture optimism in our respondents (31,32).

Children with a long-term diagnosis of epilepsy often withdraw from social situations (33). However, this was not the case during the first six months of follow-up in our study. Moreover, an encouraging finding is that children with newly diagnosed epilepsy maintained satisfactory relationships with their peers. Improving social competencies was significant despite the stigma, and both interpersonal and emotional problems. Given that children with epilepsy are interested in social contact from the very beginning, our results point to the need for their additional involvement in social activities. Due to the perceived stigma, it is crucial to work on empathy and education of their peers, as children with chronic epilepsy feel rejected and marginalized (34).

The fact that the total QOL related to epilepsy did not significantly change in the first six months is not too encouraging as the same group of patients experienced a deterioration in the general QOL (10). Potentiating the components that improve QOL related to epilepsy in the domains of interpersonal functioning is a potential preventive measure for improving the general QOL. Indeed, additional research on this topic is needed. Therefore, it is important to address the vulnerabilities in the psychosocial functioning of individuals with epilepsy in the critical period, as social and psychological support can play a pivotal role in prevention strategies (35).

Our study has several limitations. As part of a larger project that comprehensively investigates cognitive status, internalizing, and externalizing factors, and their impact on the overall quality of life, we did not delve into these aspects in this study. Also, the children were recruited from a single tertiary center possibly treating more severe cases. The picture would be more complete if more children with epilepsy in Serbia were included, although there is no official register and their total number is unknown. Future research should explore the influence of educational attainment, parental awareness, socioeconomic status, epilepsy types, specific ASM drugs, and seizure characteristics. The effects of ASM on QOL are especially relevant considering that some cross-sectional studies found that the frequency and severity of AED-related adverse effects could significantly predict the lowered levels of health-related QOL among children with epilepsy, in particular having a large impact on their psychosocial functioning (36). We hope this study inspires prospective, long-term investigations into specific domains of epilepsy-related QOL, looking for components that strengthen QOL in children with epilepsy and offering additional solutions, even in regions with medium economic development.

This study is one of the few prospective studies involving a six-month follow-up of school-age children recently diagnosed with uncomplicated epilepsy without associated comorbidities that was carried out in a medium-developed region. A noteworthy finding was a significant decline in the Interpersonal/Emotional domain and increased stigma, even with satisfactory seizure control within six months. Therefore, improving social competencies and nurturing an optimistic outlook for children with epilepsy is very important. Also, we emphasize the need for social and psychological support and the necessity of fortifying these domains and implementing preventive measures in the first six months after diagnosis.
